# Quadricuspid aortic valve with severe aortic regurgitation detected incidentally during pre-cardioversion imaging

**DOI:** 10.1093/ehjcr/ytag271

**Published:** 2026-04-21

**Authors:** Ara Baizel

**Affiliations:** Department of Cardiology, Herz-Zentrum Bodensee, Luisenstrasse 9a, 78464 Konstanz, Germany

## Case description

**Figure 1:**
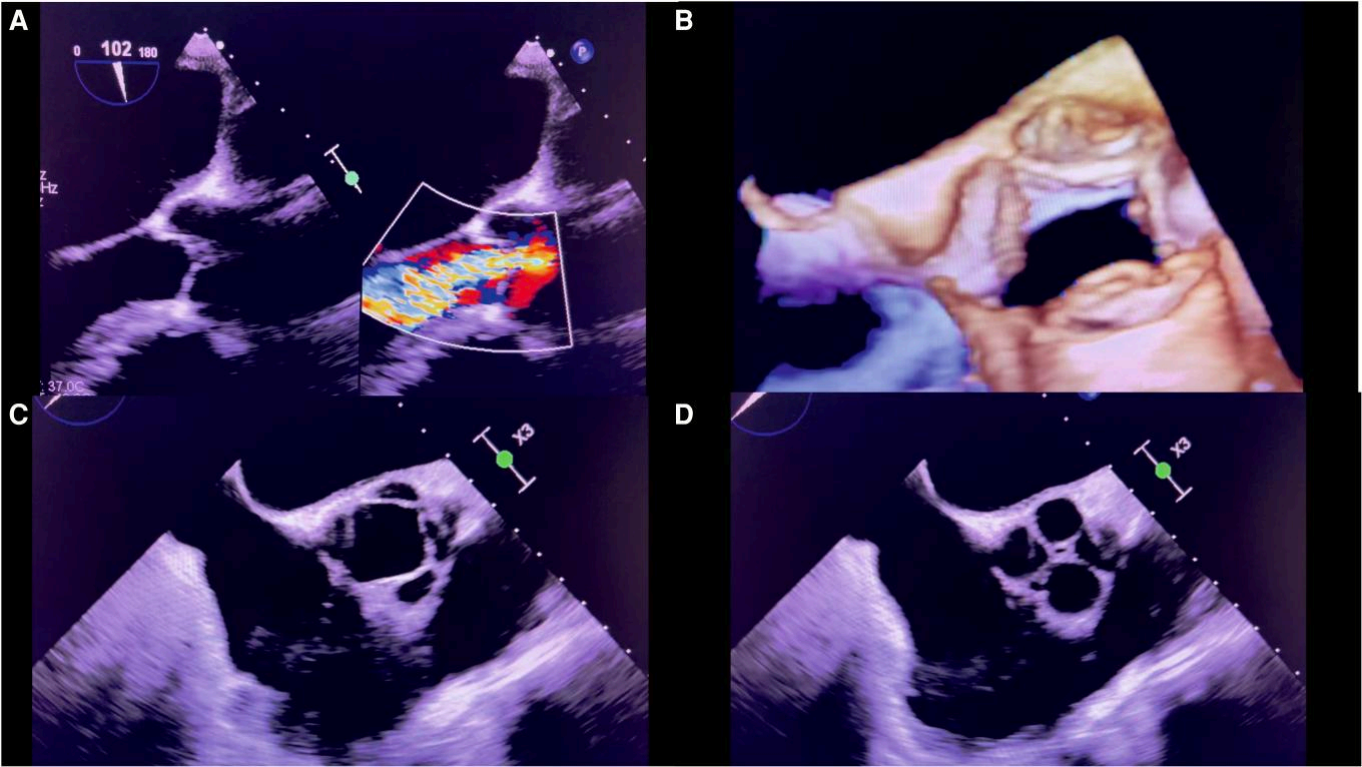
Multimodality TEE imaging of a quadricuspid aortic valve (QAV) with severe regurgitation. Panels: (*A*): Mid-esophageal long-axis view showing the regurgitant jet. (*B*): 3D TEE mid-esophageal view showing the QAV in systole. (*C*) Mid-esophageal short-axis view showing the QAV in systole. (*D*) Mid-esophageal short-axis view showing the QAV in diastole.

A 57-year-old man was referred for evaluation of newly diagnosed atrial fibrillation. Transoesophageal echocardiography (TEE), performed to exclude left atrial appendage thrombus prior to cardioversion, revealed a quadricuspid aortic valve (demonstrated in *Supplementary material online, Videos I* and *II*) with incomplete cusp coaptation and a central coaptation defect. Colour Doppler demonstrated a broad, turbulent regurgitant jet occupying most of the left ventricular outflow tract (LVOT) as demonstrated in *Supplementary material online, Video III*, directed eccentrically toward the septum and extending nearly to the apex. Late diastolic leaflet prolapse was also observed (*Figure 1A*).

Transthoracic echocardiography confirmed quadricuspid morphology and significant aortic regurgitation. Although the vena contracta measured 5 mm, additional echocardiographic parameters supported severe regurgitation, highlighting that in eccentric jets, vena contracta alone can underestimate severity due to leaflet morphology and commissural anatomy. Echocardiography also demonstrated clear signs of left ventricular remodelling, including a dilated left ventricle (LVEDD 64 mm), a more spherical ventricular shape, preserved but slightly reduced systolic function (EF 51%), and diastolic dysfunction (E/lat e′ 14). Early hypertrophy and borderline systolic function were noted, confirming ventricular remodelling.

No aortic root dilatation was observed, and computed tomography excluded associated vascular abnormalities.

Quadricuspid aortic valve is a rare congenital entity^1^ (incidence <0.05%)^2^ that frequently leads to progressive aortic regurgitation due to cusp malcoaptation. It is often detected incidentally during imaging for unrelated conditions.^2,3^ Multimodality imaging, particularly TEE, plays a key role in diagnosis by clearly visualizing cusp morphology and the mechanism of regurgitation.

Given the presence of severe aortic regurgitation, eccentric jet morphology, and early ventricular remodelling, the patient was discussed in a multidisciplinary heart team and referred for surgical valve repair.

## Supplementary Material

ytag271_Supplementary_Data

## Data Availability

The clinical data supporting the findings of this article are available from the corresponding author upon reasonable request. All data are stored in paper format and can be provided in a de-identified form to ensure patient confidentiality.
